# Combined delivery of Nogo-A antibody, neurotrophin-3 and the NMDA-NR2d subunit establishes a functional ‘detour’ in the hemisected spinal cord

**DOI:** 10.1111/j.1460-9568.2011.07862.x

**Published:** 2011-10

**Authors:** Lisa Schnell, Arsen S Hunanyan, William J Bowers, Philip J Horner, Howard J Federoff, Miriam Gullo, Martin E Schwab, Lorne M Mendell, Victor L Arvanian

**Affiliations:** 1 Brain Research Institute, University and ETH of ZurichZurich, Switzerland; 2 Northport Veterans Affairs Medical Center79 Middleville Road, Bld. 62, Northport, NY 11768, USA; 3 Department of Neurobiology and Behavior SUNY at Stony BrookStony Brook, NY, USA; 4 Center for Neural Development and Disease, Department of Neurology, University of Rochester Medical CenterRochester, NY, USA; 5 Institute for Stem Cell and Regenerative Medicine, University of WashingtonSeattle, WA, USA; 6 Department of Neurology, Georgetown University Medical CenterWashington, DC, USA

**Keywords:** motor neuron, spinal cord injury, spinal hemisection, sprouting, synaptic response, ventrolateral funiculus

## Abstract

To encourage re-establishment of functional innervation of ipsilateral lumbar motoneurons by descending fibers after an intervening lateral thoracic (T10) hemisection (Hx), we treated adult rats with the following agents: (i) anti-Nogo-A antibodies to neutralize the growth-inhibitor Nogo-A; (ii) neurotrophin-3 (NT-3) via engineered fibroblasts to promote neuron survival and plasticity; and (iii) the NMDA-receptor 2d (NR2d) subunit via an HSV-1 amplicon vector to elevate NMDA receptor function by reversing the Mg^2+^ block, thereby enhancing synaptic plasticity and promoting the effects of NT-3. Synaptic responses evoked by stimulation of the ventrolateral funiculus ipsilateral and rostral to the Hx were recorded intracellularly from ipsilateral lumbar motoneurons. In uninjured adult rats short-latency (1.7-ms) monosynaptic responses were observed. After Hx these monosynaptic responses were abolished. In the Nogo-Ab + NT-3 + NR2d group, long-latency (approximately 10 ms), probably polysynaptic, responses were recorded and these were not abolished by re-transection of the spinal cord through the Hx area. This suggests that these novel responses resulted from new connections established around the Hx. Anterograde anatomical tracing from the cervical grey matter ipsilateral to the Hx revealed increased numbers of axons re-crossing the midline below the lesion in the Nogo-Ab + NT-3 + NR2d group. The combined treatment resulted in slightly better motor function in the absence of adverse effects (e.g. pain). Together, these results suggest that the combination treatment with Nogo-Ab + NT-3 + NR2d can produce a functional ‘detour’ around the lesion in a laterally hemisected spinal cord. This novel combination treatment may help to improve function of the damaged spinal cord.

## Introduction

Several obstacles are known to prevent recovery of spinal cord function after even less-than-complete transection of the adult mammalian spinal cord. These include neurite growth-inhibiting constituents of myelin, scar-associated inhibitory factors, and the lack of sufficient neurotrophic support (Snow *et al.*, 1990; Schnell *et al.*, 1994; Tuszynski & Gage, 1995; Fawcett, 2009). Whereas treatments targeting these processes individually have proven somewhat efficacious in facilitating axon regeneration and functional recovery after spinal cord injury, the effects are generally small. Therefore, it is conceivable that developing combination treatments, e.g. neutralizing the myelin-related inhibitory molecules, combined with growth- and plasticity-enhancing factors, could be an important step in improving repair of the damaged spinal cord. This is addressed in the present study in an attempt to promote the re-establishment of a functional detour around a hemisection (Hx).

The best-studied myelin-associated inhibitory molecule is Nogo-A. Acute inactivation by function-blocking antibodies or Nogo receptor antagonists, or blockade of the downstream signals RhoA or ROCK, enhances both regeneration of lesioned fibers tracts and compensatory sprouting of the spared corticospinal tract and other fibers (Schwab, 2004; Yiu & He, 2006; Gonzenbach & Schwab, 2008). The effects of antibody-mediated neutralization of Nogo-A on ventrolateral funiculus (VLF) axons have not been studied to date.

The neurotrophic factor neurotrophin-3 (NT-3) has been shown to enhance regenerative sprouting of lesioned corticospinal, dorsal root and reticulospinal fibers in the injured spinal cord (Schnell *et al.*, 1994; Tuszynski & Gage, 1995; Bregman *et al.*, 2002; Alto *et al.*, 2009). Earlier electrophysiological studies have revealed that synaptic connections from the VLF to individual motoneurons in uninjured young (postnatal days 2–10) rats could be strengthened by administration of NT-3 (Arvanian *et al.*, 2003). However, this action of NT-3 required reversing the developmental loss of NMDA receptor activity due to Mg^2+^ block by enhancing expression of the NMDA-receptor 2d (NR2d) regulatory subunit in motoneurons using Herpes simplex virus (HSV-1) amplicon-mediated delivery of NR2d (Arvanian *et al.*, 2004).

In the current study we used a unilateral spinal cord Hx (corresponds to Brown–Sequard lesion in humans) in adult rats as a model for partial injuries because there is a clear lesion of one entire side of the cord with intact fibers remaining on the contralateral side. We examined whether intrathecal administration of a function-blocking anti**-**Nogo-A monoclonal antibody (Nogo-Ab) combined with long-term application of NT-3 (via fibroblasts) and transient facilitation of NMDA-receptor function (HSV-1-mediated viral delivery of the NMDA-nr2d subunit) could enhance re-establishment of functional synaptic connections from the transected lateral funiculi around the Hx lesion to ipsilateral lumbar motoneurons. Under these conditions novel responses were observed that differed from those in the uninjured cord in exhibiting markedly longer latency and higher electrical threshold. These positive electrophysiological findings were supplemented by anatomical studies showing long propriospinal axons crossing to the opposite side of the cord after the combination treatment. These changes were accompanied by mild improvement in recovery of motor function.

Portions of these results have been published in abstract form (Arvanian *et al.*, 2006a; Schnell *et al.*, 2007).

## Materials and methods

### Animals and experimental design

**Fig. 1:**
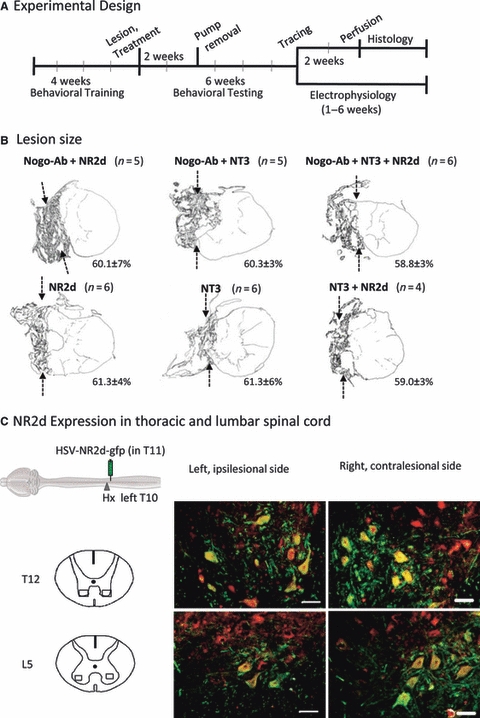
(A) Time schedule of experiments. Note that behavioral testing began before osmotic minipump removal. (B) Representative camera lucida drawing of a cross-section from a representative cord in treatment groups used for behavioral testing. The maximal lesion area of each animal was reconstructed from at least 20 spinal cord cross-sections per animal, measured with the Image J program, and expressed as percentage of the area of the T10 segment of the intact cord. Mean ± SEM value of lesion size as a percentage of an intact cord is shown for each treatment group. Arrows point to the midline. (C) Expression of GFP in motoneurons at T12 and L5 7 days after GFP-expressing HSV-1 amplicons (HSV-NR2d-GFP) were injected intraspinally at the time of a T10 lateral hemisection: GFP (as HSV-NR2d-GFP marker; red) and Peripherin (as motoneuron marker; green). Note infection of motoneurons bilaterally at T12 and L5. Further details in text. Scale bars, 50 μm.

These studies were performed in accordance with protocols approved by the Institutional Animal Care and Use Committees at SUNY/SB, University of Zurich, and Northport VAMC, USA. The design of experiments is presented in Fig. 1. A total of 126 adult female Sprague–Dawley rats (Charles River Laboratories, Wilmington, MA, USA; approximately 200 g) were housed in groups of four to six animals in standardized cages on a 12-h light–dark cycle with food and water *ad libitum*. Because of the large number of experimental groups (nine groups for the electrophysiological study, six groups for the behavioral study and five groups for the fiber tracing studies; see Results for details), the results were obtained in two separate experimental studies performed by the same investigators at two different times using the same suppliers for rats and the same treatment agents. Generally rats were pre-trained for 4 weeks to obtain baseline values in behavioral tests and then randomly divided into experimental groups according to the treatment. The rats were coded with random numbers and rats from the different groups were mixed in the cages. The experimenters were blind with regard to treatment throughout all phases of the experiment. Subsequent to surgery and treatment and in most cases behavioral evaluation, rats were used for electrophysiological recording or anatomical evaluation. However, not all treatment groups could be evaluated using all analyses (see Results). A general time line for these experiments is displayed in Fig. 1A.

### Exclusion of animals

Seven animals were excluded from the study because evaluation of the lesion *post hoc* (see Fig. 1B) revealed that it was too small or too big – in three animals we detected a portion of spared ipsilateral dorsal white matter, while in four animals overhemisection extended beyond the midline for > 10% of spared area of hemicord. Other rats were eliminated either for general health problems, especially autophagia, or because they expired during the *in vivo* electrophysiological recordings (*n* = 16).

### Surgical procedures and delivery of agents in combination treatment

In this study we used a lateral hemisection spinal cord injury model. This model allows electrophysiological evaluation of the the possibility of establishing a functional detour around the lesion. Moreover, unilateral injections of the anterograde tracer permit visualization of midline-crossing fibers rostral to the lesion and recrossing fibers caudal to the lesion (see below). Finally, transmission deficits in the chronically hemisected spinal cord coincide with clear behavioral impairments in challenging motor tasks, including irregular ladder and narrowing beam, although rats exhibit a robust recovery of their ability to walk in the open field (Arvanian *et al.*, 2009).

After pre-training on the behavioral tasks for 4 weeks, rats were deeply anesthetized and the lateral Hx was carried out at T10 as previously described (Arvanian *et al.*, 2009; Hunanyan *et al.*, 2010). Briefly, a dorsal laminectomy was performed to expose segment T10 of the spinal cord. A 1-mm slit was made in the dura at the midline at T10. A complete Hx of the left hemicord at T10 was carried out with the tip of an iridectomy scissor blade, as follows: first, a 32-gauge needle was inserted through the midline from dorsal to ventral; then one tip of the scissors was pushed along the needle through the entire thickness of the spinal cord and the left dorsal and ventral columns were cut; finally one tip of the scissors was guided along the lateral surface of the spinal cord (down to the midline) and any uncut tissue in the left lateral and ventral columns was cut.

A fine intrathecal catheter (32-gauge) was inserted from lumbar level L2/L3 and pushed up to T10 to deliver the Nogo-Ab from an osmotic minipump (Alzet^©^ 2ML2; 5 μL/h, 3.1 μg/μL) for 2 weeks. The tubing connecting the catheter with the minipump was sutured to the back muscles for stabilization. Antibody treatment was started immediately after the lesion by rinsing the wound with approximately 1 μL of the corresponding antibody. We used Nogo-Ab 11C7 (3.1 mg/mL) and monoclonal mouse IgG directed against wheat auxin as control antibody. Multiple studies have revealed the excellent distribution and penetration of anti-Nogo antibodies infused intrathecally throughout the spinal cord of adult rats and monkeys (Weinmann *et al.*, 2006). Function-blocking Nogo-Abs are also currently being applied intrathecally to spinal cord-injured patients in an on-going clinical trial (ATI-355 trial; Novartis, Basel, Switzerland).

Rat fibroblasts genetically modified to produce NT-3 (0.4 × 10^6^ cells/μL) or β-galactosidase (control) were suspended in 0.6% glucose-PBS and a cell volume of 2 μL, inserted into collagen plugs (Kawaja & Gage, 1992; McTigue *et al.*, 1998; Arvanian *et al.*, 2003) and placed on top of the lesion. These earlier studies demonstrate that this procedure results in biological effects specific to the released neurotrophin and elevation of neurotrophin levels in the spinal cord days to weeks later.

HSV-1 amplicons encoding NR2d or control β-galactosidase were administered in two injections of 1 μL each (approximately 10^4^ viral particles/μL) into the left and right ventral horn at T11 caudal to the injury region. We used a glass capillary with a tip of approximately 60 μm (calibrated for a volume of 1 μL) inserted into each side of the cord dorsum 1 mm lateral to the midline. HSV-1 amplicons have a transgene capacity sufficient to carry the NR-2d cDNA and the co-expressed green-fluorescent protein (GFP) reporter gene (Arvanian *et al.*, 2004). Previous electrophysiological studies have revealed that delivery of HSV-1 amplicon-based vectors themselves does not alter synaptic function in hippocampus (Dumas *et al.*, 1999) or spinal cord (Arvanian *et al.*, 2004, 2006b), thus supporting the use of the HSV-1 amplicon system as a safe method for delivering selected genes to the central nervous system.

To confirm the ability of HSV-1 to infect cells at a distance from the infection site, we measured GFP expression in identified motoneurons at different segmental levels. Because HSV-1 gene expression is highest 24–48 h after administration and decays to undetectable levels by 2 weeks (Bowers *et al.*, 2000), we could not use the same rats that were studied electrophysiologically or anatomically 7–12 weeks after HSV-1 administration. Therefore we used a separate control group of three hemisected rats treated in an identical manner (Fig. 1C). The degree of motoneuron infection was determined as the percentace of the total number of peripherin (green)-labeled cells also labeled with GFP (red), i.e. that were yellow. The immunolabeling procedure and analyses have previously been described (Arvanian *et al.*, 2004). We found a substantial level of infection of identified motoneurons in the vicinity of the hemisection (79% in both the T5–T7 segment (rostral to Hx) and the T11–T12 segments (caudal to Hx). Even as far caudal as the L4–L6 segments infectivity of motoneurons was 67% 7 days after injection of HSV-NR2d-GFP into the left and right ventral horn at T11 (Fig. 1C). Many other cells were also infected (GFP-labeled) at these locations but their identity as neurons or glia was not verified. These findings confirm the long-distance HSV-1 propagation within the CNS and transfer to other neurons (Zemanick *et al.*, 1991; Curanovic & Enquist, 2009), in particular motoneurons upon which both ascending and descending cells with axons in VLF have been shown to terminate, using other tracing techniques as well as electrophysiologically (Petruska *et al.*, 2007).

In order to reduce or prevent excitotoxicity that could be mediated through activation of NMDA receptors, we delivered subanesthetic doses of ketamine in all experiments (3 mg/kg, i.m., twice per day) during the initial 2 days post-injury, when transient elevation of glutamate concentration following spinal cord injury occurs (Xu *et al.*, 2004). The rationale for using ketamine, an NMDA receptor blocker known to be neuroprotective (Albers *et al.*, 1989), was to minimize glutamate-induced excitotoxicity during first 2 days after the initial lesion. In this study we did not examine the effects of ketamine alone. However, the comparisons of the effects of Nogo-Ab, HSV-NR2d and NT-3 in the various combinations were performed using the same surgical procedures and under the same recording conditions, and all animals received the same post-surgery ketamine injections.

### Behavior

The following tests were carried out. Motor tests: open-field locomotion, ladder rung walk, narrowing beam, swim tests. Sensory tests (withdrawal reflex): plantar heater, von Frey hairs. The performance of each animal was normalized to its own pre-injury baseline.

#### Open-field locomotion

This was evaluated by using the 21-point Basso, Beattie, Bresnahan (BBB) locomotor scale (Basso *et al.*, 1995). The rats were placed in an open field (diameter 150 cm) with a pasteboard-covered floor. In each testing session the animals were monitored individually for 4 min.

#### Ladder rung walk

The animals were required to walk along a 1-m-long horizontal ladder elevated to 30 cm above the ground. A defined stretch of 60 cm was chosen for filming and analysis. To prevent habituation to a fixed bar distance, the bars in this sector were placed irregularly (1–4 cm spacing). The animals performed the ladder rung walk twice in the same direction and once in the opposite direction. The number of errors (any kind of foot slip or total miss) was divided by the total number of steps in each crossing, yielding the percentage of missteps (Kunkel-Bagden *et al.*, 1993).

#### Narrowing beam

This paradigm assesses the ability of the rats to balance along a tapered beam 20 cm above the ground. The beam is flanked by two side boards and graded into 24 stretches of the same length but different widths, starting with 5 cm and ending with 1.5 cm width, and can be walked along easily by an intact animal. The maximum possible score in this test is 24. Animals had to walk along the beam three times.

#### Swim test

The setup for the swim test consisted of a rectangular Plexiglas basin (150 × 40 × 13 cm) filled with water at 23 °C. The water level was high enough to prevent the rats from touching the bottom of the basin with the tail. The animals’ task was to swim straight to the 60-cm-distant board which they could climb to reach the home cage. A total of five runs per rat was monitored using a mirror at 45° at the bottom of the pool to film the rats from the side and the bottom simultaneously. Velocity, forelimb stroke rate and inter-hindlimb coordination were analyzed.

#### Withdrawal reflex – thermal stimulation

The thermal nociceptive threshold for both hind paws was evaluated by performing a standardized plantar heater test (Hargreaves *et al.*, 1988) using a commercially available apparatus (Ugo Basile, Comerio, Italy). Rats were placed in a Plexiglas box (17 × 23 cm) and were first allowed to adjust to the new environment. When exploratory behavior ceased, an infrared source producing a calibrated heating beam (diameter 1 mm) was placed under the hind paw and triggered together with a timer. After one initial trial, the time for the hind limb withdrawal reflection was averaged from four successive measurements. A minimum interval of 30 s was maintained between successive trials.

#### Withdrawal reflex – mechanical stimulation

Von Frey hairs (Semmes-Weinstein Monofilaments; Stoelting Co., Wooddale, IL, USA) with target force ranging from 0.008 to 300 N were used. Rats were placed in a Plexiglas box (17 × 23 cm) with a fine grid bottom and were first allowed to adjust to the new environment. The monofilament was pressed against the plantar surface of the foot at a 90° angle until it bowed, and held in place for 1–2 s. This stimulation was repeated up to three times in the same location. The test was performed by using increasing filament calibers until the first withdrawal reflex was noted.

### Tracing

We assessed the crossing of propriospinal fibers that would project through the VLF as this was the tract that was activated electrophysiologically. Biotin dextran amine (BDA; 10%, MW 10 000) in a total volume of 1.0 μL was injected unilaterally into four sites in the left ventral horn at C4–C7 over a period of 10 min for anterograde tracing of midline-recrossing fibers. Ten days later the rats were perfused and spinal cords were removed and prepared for morphological evaluation. Alternating coronal sections were processed with Cresyl violet or were stained for BDA using a nickel-enhanced diaminobenzidine protocol. The number of fibers originating from the gray matter at C4/C7 and traced with BDA was analyzed quantitatively using a light microscope with bright-field illumination. We assessed the numbers of midline-crossing fibers above and below the lesion in every fourth (30-μm-thick) cross-section from the entire spinal cord (i.e. in approximately 500 sections per cord). For normalization, all midline-crossing fibers were counted from T11 to S5 (below the lesion) and standardized to the number of crossing fibers at T8 (above the lesion).

### Electrophysiology

Experimenters were blinded as to the treatment of each rat. Rats were deeply anesthetized using i.p. injection of ketamine (80 mg/kg, 0.5 mL) and xylazine (10 mg/kg, 0.5 mL). Heart rate and expired CO_2_ were monitored continuously. Dorsal laminectomy of the spinal cord was performed at T6–T8 for placement of the stimulation electrode and L1–L6 for placement of the recording electrodes. L1–L6 ventral spinal segments were held tightly between custom-made bars, and the dorsal surface of the cord was imbedded in a 3-mm-thick agar layer to minimize movement of the cord during recordings. We recorded responses from L5 motoneurons below the lesion (T10) on the same side as the Hx. These responses were evoked by stimulation of ventrolateral white matter tracts at T6 on the same side of the cord (details in Arvanian *et al.*, 2009). Motoneurons were identified by the antidromic response to stimulation of the cut L5 ventral root. The resting membrane potential of motoneurons used for analyses ranged from −55 to −65 mV. Peak excitatory postsynaptic potential amplitude was measured from pre-stimulus baseline to peak. Latency was measured from stimulus artifact to response onset. After completion of electrophysiological recording, the rats were perfused and spinal cords removed and prepared for morphological evaluation of the injury level.

### Statistics

For the behavior experiments, two-way repeated-measures anova and pairwise multiple comparison procedures (Holm–Sidak method) were used to determine the statistical significance of the results (*P* < 0.05). Data from the tracing experiments were subjected to one-way anova followed by Bonferroni's *post hoc* pairwise comparisons (*P < 0.05). For the electrophysiological studies, the mean maximum response from each motoneuron (50 consecutive responses per cell) was averaged over all motoneurons recorded in each rat and these averages were compared between treatment groups using one-way anova or one-way anova on ranks (means are expressed ± SEM; *n* = number of rats). If significant differences were observed between groups, a Student–Newman–Keuls test or Dunn's method were used for pairwise comparisons as appropriate.

## Results

### Electrophysiology

The goal was to determine whether the combination treatment induced the appearance of new functional connections spanning the hemisected segment. We recorded intracellularly from motoneurons below the lesion ipsilateral to the Hx. Responses were evoked by stimulation of the ipsilateral VLF white matter above the lesion. This approach improves detection of very weak functional connections across the injury region and enables investigation of the impact of the various treatments on these connections. For electrophysiology experiments we used nine groups: one non-injured group that received all control treatments, and eight groups that received a Hx lesion and no treatment, or treatment with one, with two, or with all three components of the combination treatment; appropriate controls were administered in cases where only one or two active components were delivered. The results are from experiments conducted 7–12 weeks after the surgery with different treatment groups randomly assigned to these times in order to minimize the variability of post-operation recording time among the groups (Fig. 1).

#### Hx disrupted monosynaptic connections to motoneurons and additive treatments established novel polysynaptic connections

**Fig. 2:**
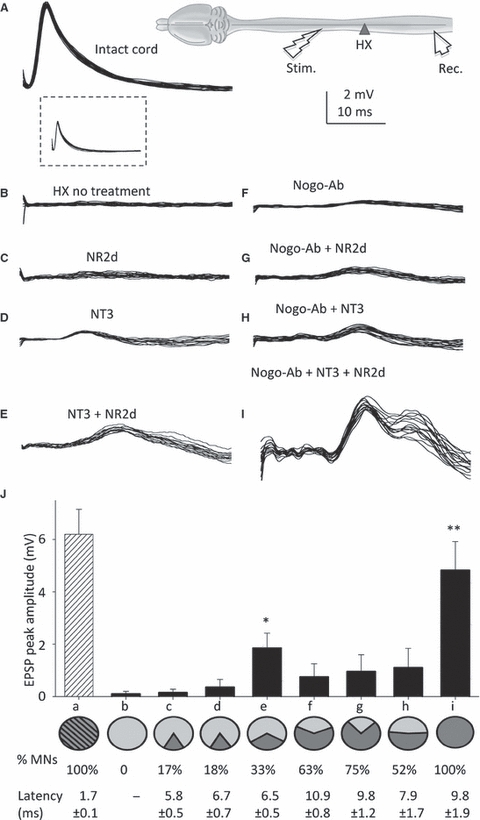
Intracellular recordings from L5 motoneurons demonstrating that Hx lesion abolished monosynaptic transmission and additive Nogo-Ab + NT-3 fibroblasts +HSV-NR2d treatment established strong polysynaptic responses from ipsilateral VLF rostral to Hx. (A) Superimposed consecutive traces demonstrating monosynaptic responses in non-injured cord with control treatments (*n* = 56 cells in seven rats). (B–I) Polysynaptic responses in Hx cords with following treatments: (B) control-Ab+ control-FB+control-HSV (*n* = 35 cells in five rats; inset: response of same motoneuron, but from ipsilateral VLF caudal to Hx); see schematic position of recording and stimulating electrodes for this particular configuration; (C) control-Ab+control-fibroblasts +HSV-NR2d (*n* = 31 cells in six rats); (D) control-Ab + NT-3 fibroblasts +control-HSV (*n* = 39 cells in six rats); (E) control-Ab + NT-3 fibroblasts +HSV-NR2d (*n* = 65 cells in 10 rats); (F) Nogo-Ab+control-fibroblasts+control-HSV (*n* = 41 cells in seven rats); (G) Nogo-Ab+control-fibroblasts +HSV-NR2d (*n* = 29 cells in six rats); (H) Nogo-Ab + NT-3 fibroblasts+control-HSV (*n* = 32 cells in six rats); (I) Nogo-Ab + NT-3 fibroblasts +HSV-NR2d (*n* = 76 cells/10 rats). (J) Summary of results for A–I groups, respectively; pie charts represent the percentage of responding motoneurons in each group. Top drawing shows a schematic position of recording and stimulating electrodes (except inset in B). *P < 0.05 for the effect of NT-3 + NR2d treatment compared to all groups, except full treatment group. **P < 0.01 for the effect of full treatment group vs. all other groups.

In uninjured control rats that received laminectomy and treatments with controls for all three agents in the combination treatment (Ringer-filled catheter, control fibroblasts, and control HSV-1 virus), the response in L5 motoneurons from ipsilateral T6 VLF exhibited the following properties: large peak amplitude (6.2 ± 0.8 mV), short latency (1.7 ± 0.1 ms), brief rise time and minimum fluctuation in both amplitude and latency (Fig. 2A; *n* = 56 cells from seven rats). These responses reached maximum amplitude at relatively low stimulus current intensity (67.8 ± 11.5 μA, 50 μs), were similar to those recorded in L5 motoneurons from ipsilateral VLF in untreated intact adult rats (Arvanian *et al.*, 2009), and were probably monosynaptic.

In Hx-lesioned rats that received either no treatment or control treatment, the mean response was barely distinguishable from baseline [no treatment: mean 0.2 ± 0.3 mV, *n* = 7, not shown; control treatment (Fig. 2, B), mean 0.1 ± 0.2 mV; *n* = 5], even with VLF stimuli as intense as 600 μA at 50 μs width. When we repositioned the stimulation electrode caudal to the lesion, a typical monosynaptic response was recorded from the same motoneuron at low stimulus intensity (Fig. 2B inset). These results indicate that motoneurons below the lesion remained viable and capable of receiving inputs from surviving propriospinal fibers in the VLF below the lesion, and that the lack of transmission from above the lesion was due to a disrupted connection.

**Fig. 3:**
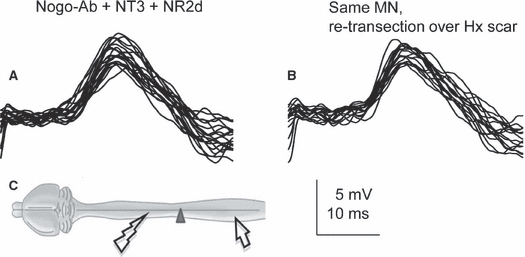
(A) Stimulus to VLF rostral to a hemisection evoked a response in an ipsilateral motoneuron caudal to the hemisection in a rat treated with Nogo-Ab + NT-3 + NR2d. (B) Recording from the same motoneuron after a re-transection of the spinal cord through the Hx area. (C) Diagram to show position of the recording and stimulating electrodes. Note that retransection did not eliminate the response to VLF stimulation.

The striking finding was that the additive treatment (Nogo-Ab + NT-3 + NR2d) induced the appearance of large (4.7 ± 1.2 mV, *n* = 10 rats; Fig. 2I) responses in all (100%) injured rats. However, in contrast to the short-latency monosynaptic responses in uninjured rats, these responses exhibited a long latency (9.8 ± 1.9 ms), showed greater fluctuation in both amplitude and latency (Fig. 2, A vs. I), and required a markedly higher stimulus intensity (415 ± 53 μA, 50 μs) to evoke a maximum response. These results suggest that the functional connections established in the Hx-lesioned spinal cords that received the combination treatment with Nogo-Ab + NT-3 + NR2d probably involved conduction in smaller axons than those responsible for the responses in intact preparations, required more spatial summation on interneurons and were probably multisynaptic. In rats treated with each agent alone or in pairs [with corresponding controls for the missing agent(s)], the multisynaptic responses evoked from segments above the Hx were either absent or were much smaller than in rats with the full combination treatment, even at the high stimulus currents (600 μA, 50 μs). Treatment with Nogo-Ab alone (0.8 ± 0.5 mV; Fig. 3F), or in combination with either NT-3 (1.1 ± 0.7; Fig. 3H) or NR2d (1.0 ± 0.7 mV; Fig. 3G), resulted in weaker multisynaptic connections that could be recorded in over half the rats (52–75%). Treatment with NT-3 + NR2d induced the appearance of larger responses (peak amplitude 1.9 ± 0.7 mV; Fig. 2E) but did so in only a few rats (in three rats out of 10 studied). In rats that received NR2d alone (0.2 ± 0.1 mV; Fig. 2C) or NT-3 alone (0.4 ± 0.3 mV; Fig. 2D) very weak connections in a small subset of rats were noted. Reconstruction of the Hx in each case revealed no relation between the size of the lesion and the amplitude of the responses (but see Discussion).

#### Novel polysynaptic responses were the result of a ‘functional detour’ around the Hx lesion

It was important to determine whether novel polysynaptic connections established in the triple combination group travelled through the lesion area or around the Hx. Therefore, in three rats treated with the full combination treatment, the spinal cord was carefully retransected through the existing scar after recording polysynaptic responses from several motoneurons while maintaining penetration of a motoneuron. We found that these responses persisted after this procedure (Fig. 3). These results confirm that the novel responses recorded in Hx-lesioned rats receiving the full combination treatment were the result of the establishment of new connections around the hemisected cord rather than regeneration through the lesion area.

### Anatomical evaluation

**Fig. 4:**
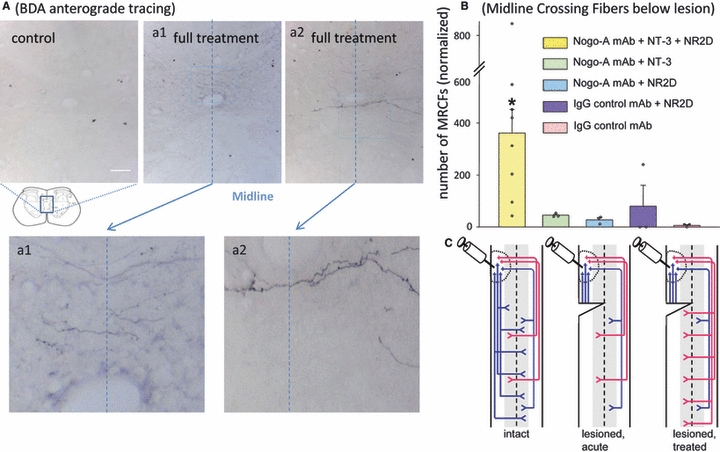
Combination treatment enhanced ‘anatomical plasticity’ following Hx injury. (A) Representative photomicrographs of 30-μm cross-sections below the lesion to demonstrate midline-recrossing fibers (MRCF) revealed by BDA anterograde transport from the gray matter at C4-C7. Further details in text. Left panel shows example from animal treated with control-Ab and schematic depiction of the area shown. Only very few fibers could be found. Middle and right panels showed MRCF in two different animals treated with Nogo-Ab + NT-3 + NR2d. (B) Bar graph of normalized number of re-crossing fibers counted from T11 – L5. The number of crossing fibers at the T8 level (above the lesion) was used to normalize these results for differences in staining intensity. Results of individual experiments displayed as dots in line with the bars. Some points overlap. (C) Schematic drawing of the labeling in unlesioned animals (left panel), the effect of the lesion on ipsilateral fibers (middle panel) and the regenerative sprouting effect (right panel) after the combination treatment with Nogo-Ab + NT-3 + NMDA2d. Blue fibers cross the midline just once, and red fibers cross the midline twice (MRCF). Scale bar in A, 100 μm (does not apply to insets from a1 and a2, which are at higher power for visualization of thin and thick fibers, respectively). * denotes treatments with more recrossing fibers than IgG control- treated preparations.

The primary goal was to determine a possible anatomical substrate for the novel polysynaptic responses around the Hx in the triple combination treatment which gave the most robust change in the electrophysiological studies. We were particularly interested in determining whether the electrophysiological changes are related to an increase in the number of midline crossings of fibers. Anterograde BDA tracing from injections at C4/C7 ipsilateral to the Hx was carried out in order to assess the number of fibers that crossed the midline caudal to the Hx (Fig. 4A). This injection site allowed us to follow the projection of interneurons (i.e. long propriospinal neurons; Reed *et al.*, 2008) to the side contralateral to the Hx and back to the lesioned side more caudally. Above the lesion, fibers crossing the midline to the contralesional side cannot be distinguished from fibers that re-cross to the ipsilesional side but below the lesion only midline-recrossing fibers are labeled (see Fig. 4C).

Because each group consisted of 500 sections from each of three to eight cords, it was impractical to study all eight groups. Instead we compared the results of animals treated with all three agents (Nogo-Ab + NT-3 + NR2d; *n* = 8 rats after exclusion) to results obtained from an untreated hemisected group (IgG control mAb; *n* = 3 rats after exclusion) which displayed no electrophysiological evidence of a functional detour. We made similar determinations using rats treated with two agents (Nogo-Ab with either NT-3 or NR2d) that gave intermediate electrophysiological evidence of a detour, or NR2d alone that produced a minimal detour. In the unlesioned cord the number of crossing fibers between T11–L5 was very high (approximately 5000; *n* = 3). In Hx cords the number of crossing fibers above the lesion was also high, and we used the number of fibers at T8 to normalize for tracer injection quality. Counts of these crossing fibers (Fig. 4B) revealed that the group of animals with the combined Nogo-Ab + NT-3 + NR2d treatment was unique in having appreciable numbers of re-crossing fibers (362 ± 90.1 SEM, *n* = 8 rats). This was significantly higher (*P* < 0.05) than the number observed in the other treated Hx groups. Administration of control-Ab alone yielded counts of recrossing fibers that were uniformly very low. However, in individual experiments in the three groups with intermediate treatments we observed some recrossing fibers in all preparations treated with anti-Nogo and NT-3 or NR2d and this might account for the electrophysiological evidence of the detour (Fig. 4B). In preparations treated with NR2d only we observed no recrossing fibers in three animals; this is consistent with the lack of detour observed electrophysiologically. One preparation had an anomalously high number of recrossing fibers.

In agreement with the electrophysiological findings above, where no monosynaptic response in motoneurons was found in response to stimulating VLF rostral to the Hx and where a re-transection of the spinal cord did not alter the polysynaptic response, we did not find any evidence for axons that could have crossed through the lesion. We therefore consider it likely that the conduction path involves newly formed re-crossing fibers from long propriospinal axons, which connect via interneurons to the L5 motoneuron pool in the ventral horn of the ipsilesional side (Fig. 4D; see Discussion).

### Assessment of lesion size

After completion of physiological recordings or tracing experiments the lesion site was reconstructed from cross-sections and measured as a percentage of the area of the intact cord (Fig. 1B). Camera lucida drawings of six representative lesion sites from rats used for the behavioral studies are shown in Fig. 1B. It can be seen that the mean lesion size was virtually identical for all treatments. There were differences in the tissue that was spared from rat to rat but these were not systematic in the different treatments (see Discussion).

### Behavioral evaluation

In order to minimize the role of extraneous factors, it was important to carry out behavioral testing on animals that arrived at the institutional animal facility from the same vendor at the same time and received treatment using the same lot of compounds. The need to do surgery and behavioral testing on a single group of animals placed a limit on the number of animals that could be studied. Thus we limited this experiment to six groups (vs. nine groups in the electrophysiology experiment). The groups were chosen to cover the range of results obtained in the initial electrophysiology experiments. All rats were pre-trained for 4 weeks, then received injury and treatment within 4 days and behavioral testing for the following 6 weeks using four motor and two sensory tests.

**Fig. 5:**
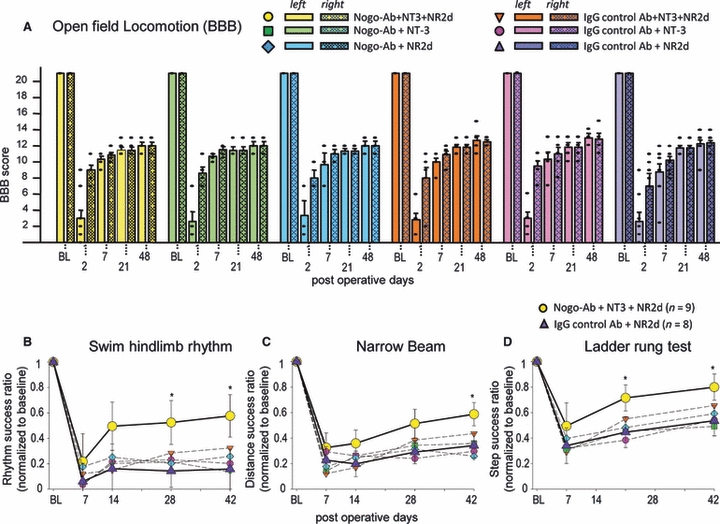
Effects of treatment on behavioral outcome. (A) Open-field locomotion revealed similar scores in all six groups at 2 days post-operation, indicating consistency of the lesion. However, there were no significant differences among the groups during up to 48 days post-injury. (B–D) In more challenging behavioral tests, i.e. swim, narrow beam and ladder rung, the full triple-treatment group showed better recovery than animals of other groups (performance of each animal was normalized to its own pre-injury baseline). As performance of all groups that received one or two treatment components was similar, the mean error bars are displayed for the NR2d-alone and the triple combination groups. *denotes times at which the full treatment group was significantly different (*P* < 0.05) from the partially treated groups.

Two days after the operation, the rats were scored with the BBB test (Basso *et al.*, 2002) to assess the extent of the lesion. The left leg did not score > 3 in any group while the right hindlimb was mostly able to support the body weight and perform plantar stepping, which is equivalent to a score of 8 or higher. In the course of a 3-week recovery the different groups improved their performance gradually by approximately 3–4 points and reached a plateau of 12 points (which is just below the score for coordinated forelimb–hindlimb stepping), with no significant difference between the groups (Fig. 5A). Although the quasi-quantitative protocol of BBB scoring is useful for evaluating the loss of function and recovery following injury, it has a major disadvantage in assessing the subtle improvements that result from treatments after thoracic Hx in rodents because of the robust spontaneous recovery of locomotor function that takes place after this type of injury (Courtine *et al.*, 2008; Arvanian *et al.*, 2009). Therefore all animals were also tested in more challenging tests such as the symmetry of swimming, narrowing beam and horizontal ladder paradigms (Fig. 5B and C; see below).

In the swim test there was no difference in swimming speed between the groups (*P* > 0.05), as the Hx lesion allowed a relatively fast recovery of performance in this weight-supported test. We therefore focused on inter-hindlimb rhythm and measured the difference in beat duration (time for a complete stroke) between the right and the left hindlimb. In healthy, uninjured animals this difference is close to zero as the legs beat in a very regular pattern. After a lateral Hx lesion the animals exhibited ‘limping’, which is better observed and measured during swimming than in overground locomotion (BBB test). This interhindlimb coordination remained disturbed over the entire 42-day period following the lesion, but the difference in beat duration was smallest in the triple-combination treatment group (Fig. 5B). While the performance among the groups with one or two treatment components was similar (*P* > 0.05), the performance of the rats from the triple combination treatment group was significantly better than these other groups (*P* < 0.05).

In the narrow beam test, unlesioned animals normally reach the narrow end of the scaled bar without missteps. After the lesion, this capability was greatly reduced. Normalized values revealed that the triple combination treatment group performed significantly better than groups with one or two treatment components (*P* < 0.05). The performance among groups with one or two treatment components was similar (*P* > 0.05).

In the horizontal ladder rung test, the ability of the animals to place their hindpaws on the same rung as the forepaws was greatly reduced on the ipsilesional side, where hardly any successful hindpaw placements were performed (Fig. 5D). Significantly better performance was evident in the triple combination treatment group compared to the other groups tested at post-operative day 28; this difference was maintained at day 42, the last time point tested (*P* < 0.05). The performance among groups with one or two treatment components was similar (*P* > 0.05).

**Fig. 6:**
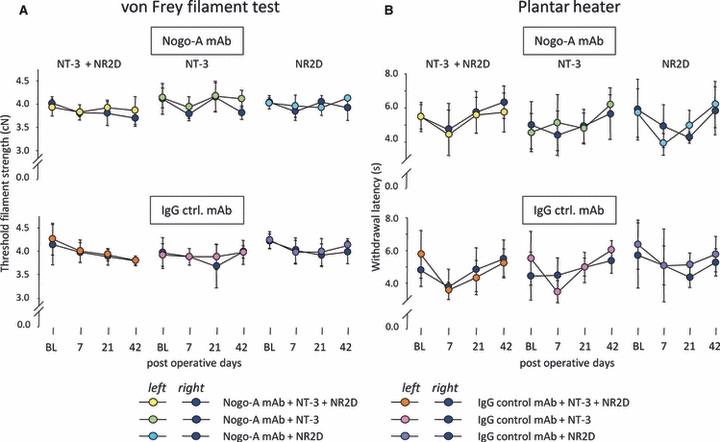
Sensory tests. (A) The von Frey filament test revealed no difference in mechanical threshold between the hind paws of the lesioned and unlesioned side in all groups. (B) Withdrawal latency to noxious thermal stimulation was not different between hind paws on the intact and hemisected side.

In order to evaluate the effects of the treatment on the sensitivity to nociceptive stimuli, we performed standardized von Frey filament and plantar heater tests. Over the 7, 21 and 42 days post-operation time points tested, treatment groups were indistinguishable from each other (Fig. 6).

## Discussion

This study has revealed that a functional ‘detour’ can be established around a Hx, from the lesioned ventrolateral white matter above to ipsilateral motoneurons below, using a novel combination treatment with the following components: an antibody to the major inhibitory molecule Nogo-A, the neurotrophin NT-3, and the NR2d regulatory subunits to enable NT-3-induced plasticity. Combining neurotrophins with other agents to improve their effectiveness in restoring function is consistent with the results of recent studies adopting this approach (Lu *et al.*, 2004; Nothias *et al.*, 2005; Arvanian *et al.*, 2006b; Chen *et al.*, 2008; Massey *et al.*, 2008). Furthermore, the VLF contains reticulospinal and long propriospinal fibers (Reed *et al.*, 2008) known to participate in the recovery of locomotor function in rats following thoracic injuries (Basso *et al.*, 2002; Schucht *et al.*, 2002; Arvanian *et al.*, 2009). Therefore, development of a treatment aimed at the restoration of VLF projections is an important strategy in promoting recovery of function after thoracic injuries.

Electrophysiological experiments revealed the appearance of novel long-latency responses in L5 motoneurons from the ipsilateral VLF rostral to Hx in rats receiving the combination treatment (Fig. 2). The fact that these responses were preserved after re-transection of the spinal cord through the pre-existing lesion strongly suggests that these novel responses were not due to axons regenerating through the lesion, but were the result of the establishment of novel functional connections around the Hx. Although the increase in branching from white-matter fibers observed in the tracing studies is suggestive of new connections being responsible for the detour, we cannot rule out a contribution from already existing subliminal connections or ‘silent’ synapses (Kerchner & Nicoll, 2008) that were strengthened and became visible after the treatment (Wall, 1988).

Previous double-labeling studies in intact adult rats using tracers injected into the lumbar cord and VLF have identified a population of cervical neurons that cross to the contralateral VLF and recross to terminate in the ipsilateral upper lumbar cord (Reed *et al.*, 2008). However, the absence of electrophysiological responses observed in control hemisected preparations suggests very little functional connectivity to L5 motoneurons mediated by propriospinal fibers crossing above and below the Hx (Fig. 4). The propriospinal fibers studied here apparently did not sprout below the lesion spontaneously, as indicated by the virtual absence of midline-recrossing fibers below the Hx in the absence of treatments. However, after the full combination treatment the number of fibers recrossing caudal to Hx increased substantially (Fig. 4).

The mild behavioral effects of the combination treatment occurred on a background of robust spontaneous recovery of locomotor function observed after thoracic Hx in rodents (Courtine *et al.*, 2008; Arvanian *et al.*, 2009); this spontaneous recovery makes further improvements difficult to detect. More challenging tests such as narrowing beam, horizontal ladder and the swimming symmetry revealed minor yet significant improvement of motor function in rats with the full combination treatment (Fig. 5), with no change in nociceptive function (Fig. 6). More robust recovery may depend on strengthening the synaptic connectivity from the descending fiber systems on the hemisected side to neurons responsible for the detour.

Together these studies suggest that the combination treatment produced larger effects electrophysiologically, anatomically and behaviorally than components tested separately or in pairs. In the electrophysiology where all possible combinations were tested with corresponding controls, the ability of these agents to produce a detour is clear. In the case of the anatomy and behavior, the full combination treatment elicited more sprouting or functional recovery than any of the treatments tested. However, because not all combinations were studied anatomically and behaviorally, and because the behavioral recovery may not parallel the electrophysiological recovery, we remain cautious about making conclusions concerning the ability of these treatments to promote recovery of behavior. Another caveat is the possibility of small differences in tissue sparing among the different preparations (Fig. 1B). However, the uniform differences in plasticity at all levels between the treatment groups, and the uniformity of the lesion size, make it very unlikely that systematic differences in tissue sparing were a major factor determining the findings reported here.

How does the combination treatment produce the detour? The requirement for Nogo-A specific antibody and NT-3 and NR2d suggests that detour formation required sprouting or growth of axons as well as an increase in synaptic efficacy. Although our present results do not reveal the location of the novel connections, we believe that they are distributed throughout the cord but are probably most numerous or strongest close to the lesion site where the concentration of the exogenous agents is highest.

NMDA receptors on motoneurons become functional in the prenatal period (Ziskind-Conhaim, 1990; Kalb & Hockfield, 1992), but they suffer a decline in function during the second postnatal week due to Mg^2+^ blockade (Arvanian *et al.*, 2004). We previously found that restoring NMDA receptor function by adding back the NR2d subunit of the NMDA receptor using an HSV viral construct enabled NT-3 to induce NMDA receptor-dependent potentiation of VLF synaptic transmission (Arvanian *et al.*, 2004). When combined with NT-3, the NR2d subunit induced the appearance of synaptic responses in motoneurons from damaged VLF axons (Arvanian *et al.*, 2006c). These results suggest that activity of NMDA receptors in the target neurons might be an essential factor required for growing axons to establish glutamatergic synaptic contacts upon them. Although our studies were in motoneurons, it seems likely that novel connections on interneurons are important for establishing the connections to motoneurons described here.

Our current results demonstrate that combinatorial treatment with NT-3 and NR2d resulted in VLF connections in approximately 33% of the motoneurons in injured adult rats. Similar treatments with NT-3 and NR2d in contused or staggered double-hemisected neonatal rats resulted in recovery of some connectivity to virtually all motoneurons (Arvanian *et al.*, 2006c). One possible explanation for the limited efficacy of the treatment with NT-3 and NR2d in adult rats is the age-related development of myelin-associated neurite growth inhibition in the spinal cord. The localization of Nogo-A in oligodendrocytes, where expression starts at a relatively late developmental stage (Huber *et al.*, 2002; Taketomi *et al.*, 2002), fits well with its role as an age-dependent myelin-associated inhibitor of regenerative fiber growth in adult mammals. Here we demonstrate that additive treatment with NT-3 and HSV–NR2d in adult rats was sufficient to form connections via conduction around the Hx only when combined with anti-Nogo-A antibody. Considering that HSV-1-mediated NR2d expression lasts 1–2 weeks and Nogo-Ab delivery lasts 2 weeks, we hypothesize that they play a role in initiating the establishment of polysynaptic connections observed 7–12 weeks post-injury.

The establishment of connections to motoneurons via the detour is supported by anatomical experiments that indicate an increase in the number of branches given off by propriospinal or supraspinal axons (either ascending or descending) in the contralateral white matter caudal to Hx. Growth of these branches to the ipsilesional side of the cord could provide access to the stimulating electrode above the lesion; similarly, the recrossing between the hemisection and L5 could provide access to ipsilesional motoneurons, perhaps via strengthening of polysynaptic connections. Branches of fibers ipsilateral to the Hx may also send branches to contact propriospinal neurons on the contralateral side which then recross below the Hx to contact L5 motoneurons either directly or via relays from short propriospinal interneurons (Courtine *et al.*, 2008; Etlin *et al.*, 2010). Axotomized fibers descending from supraspinal centers (Reed *et al.*, 2008) including the corticospinal tract and serotonergic raphe spinal fibers known to be influenced by anti-Nogo (Liebscher *et al.*, 2005; Müllner *et al.*, 2008) could also play a role in re-establishing the connectivity observed in these experiments.

A further consideration is the recent finding that thoracic Hx can reduce conduction through the uninjured contralateral white matter beginning 1–2 weeks after Hx as well as a decline in conduction velocity for axon segments across from the Hx (Arvanian *et al.*, 2009). These changes were associated with decreased excitability in these axons (manifested by an increased rheobase), partial demyelination of the VLF and rubrospinal tract axons contralateral to the Hx (Hunanyan *et al.*, 2011) and accumulation of chondroitin sulfate proteoglycans (CSPGs) in tissue surrounding the Hx (Hunanyan *et al.*, 2010). Such changes undoubtedly contributed to the absence of any response through the region of injury in the controls; the treatments given at the time of the injury could have either prevented this decline in conduction or reversed it. In this context, NT-3 has been found to induce oligodendrocyte proliferation and myelination of regenerating axons in the contused adult rat spinal cord (McTigue *et al.*, 1998), and the presence of Nogo has complex effects on oligodendrocyte differentiation which could affect myelination and impulse conduction (Pernet *et al.*, 2008). The effects of NT-3 and anti-Nogo on myelination of regenerating fibers and conduction through the region contralateral to Hx, as well as the combination of this treatment with intraspinal digestion of CSPGs (Fawcett, 2009), remain to be determined in the current Hx model.

In conclusion, these results demonstrate that combination treatments using anti-Nogo, NT-3 and the NR2d subunit promote the establishment of a synaptic detour around a Hx. This pathway involves sprouting of white-matter fibers to the opposite side and may contribute to behavioral improvement. Future experiments should explore this combination approach to studying other spinal injuries, e.g. contusion, to determine whether recovery of function is improved under these experimental conditions.

## Abbreviations

BDA: biotin dextran amine
GFP: green-fluorescent protein
HSV-1: Herpes simplex virus
Hx: hemisection
Nogo-Ab: anti**-**Nogo-A monoclonal antibody
NR2d: NR2d subunit of NMDA receptor
NT-3: neurotrophin-3
VLF: ventrolateral funiculus
